# Multi-view feature representation and fusion for drug-drug interactions prediction

**DOI:** 10.1186/s12859-023-05212-4

**Published:** 2023-03-14

**Authors:** Jing Wang, Shuo Zhang, Runzhi Li, Gang Chen, Siyu Yan, Lihong Ma

**Affiliations:** 1 School of Computer and Artificial Intelligence, Zhengzhou University, Zhengzhou, China; 2 Cooperative Innovation Center of Internet Healthcare, Zhengzhou University, Zhengzhou, China; 3 School of Artificial Intelligence, Henan University, Zhengzhou, China; 4 Fuwai Hospital, National Center for Cardiovascular Diseases, Chinese Academy of Medical Sciences and Peking Union Medical College, Beijing, China

**Keywords:** Drug-drug interactions, Graph representation, Drug molecular structure, Semantic information extraction, Feature fusion

## Abstract

**Background:**

Drug-drug interactions (DDIs) prediction is vital for pharmacology and clinical application to avoid adverse drug reactions on patients. It is challenging because DDIs are related to multiple factors, such as genes, drug molecular structure, diseases, biological processes, side effects, etc. It is a crucial technology for Knowledge graph to present multi-relation among entities. Recently some existing graph-based computation models have been proposed for DDIs prediction and get good performance. However, there are still some challenges in the knowledge graph representation, which can extract rich latent features from drug knowledge graph (KG).

**Results:**

In this work, we propose a novel multi-view feature representation and fusion (MuFRF) architecture to realize DDIs prediction. It consists of two views of feature representation and a multi-level latent feature fusion. For the feature representation from the graph view and KG view, we use graph isomorphism network to map drug molecular structures and use RotatE to implement the vector representation on bio-medical knowledge graph, respectively. We design concatenate-level and scalar-level strategies in the multi-level latent feature fusion to capture latent features from drug molecular structure information and semantic features from bio-medical KG. And the multi-head attention mechanism achieves the optimization of features on binary and multi-class classification tasks. We evaluate our proposed method based on two open datasets in the experiments. Experiments indicate that MuFRF outperforms the classic and state-of-the-art models.

**Conclusions:**

Our proposed model can fully exploit and integrate the latent feature from the drug molecular structure graph (graph view) and rich bio-medical knowledge graph (KG view). We find that a multi-view feature representation and fusion model can accurately predict DDIs. It may contribute to providing with some guidance for research and validation for discovering novel DDIs.

## Introduction

Drug-drug interactions (DDIs) are changes in the interactions among drugs taken simultaneously or continuously [1, 2]. In general, DDIs mainly include pharmacokinetic interactions and pharmacodynamics interactions. It is an extremely complex process to verify drug interactions among drugs in pharmacology. Clinically, DDIs is a double-edged sword. Firstly, it has beneficial effects. DDIs could not only improve efficacy and reduce adverse effects among drugs, but also provide relief from drugs poisoning and prevent the development of drugs resistance. For example, when caffeine is combined with ergotamine, solubility is increased, absorption is increased and efficacy is improved. It is important to pay attention to drugs interactions in order to improve the quality of care and the safe and effective use of combination drugs. Meanwhile, DDIs may cause adverse drug interactions (ADRs), and literature [3] gives the ADRs rates. The rate reaches 100% when at least 6 kinds of drugs are taken simultaneously. Obviously, when a drug is co-administered with another and multiple drugs, it will cause many ADRs that may increase morbidity and mortality. Thus, identifying potential DDIs as much as possible is vital for safer and improved patient prescriptions [4]. Recently, many models have been developed on DDIs prediction. And the basic methods are the traditional laboratory-based models, which are time-consuming and costly [5], causing the limitation of the ability to discover potential DDIs. Thus computational approaches provide practical strategies for predicting DDIs. They mainly include machine learning-based models and deep learning-based models.

For machine learning-based models, Most of them adopt integrating more data sources to capture drug properties, including similarity features, such as molecular structure [6–8], side effects [9, 10] and genomic similarity [11]. These methods rely on handcrafted features and domain knowledge. To alleviate this phenomenon, deep learning-based models gradually prevail, obtaining abstract features instead of handcrafted features. However, some works [12–15] only focus on the structure information or SMILES sequences [16] of drugs but ignore the rich semantical information related to drugs. Others [17–19], on the contrary, use knowledge graph (KG) to capture the rich bio-medical information but ignore the molecular structural feature of drugs.

Although the above models have achieved good performance, they simply consider the drug structure information or the rich semantic feature brought by the knowledge graph to determine the final feature representation of drugs, thus limiting its predictive capability. Moreover, these methods mainly explore binary DDIs prediction, however, it is more valuable but challenging to predict multi-typed DDIs. MUFFIN [20] is proposed to achieve both binary DDI prediction and multi-class DDI prediction, which considers both drug molecular structure and rich semantic features in KG. Inspired by MUFFIN, this work not only extracts features from drug molecular structure but also considers the topological feature from bio-medical KG. However, MUFFIN cannot distinguish different graph structures based on the drug molecular structure and lacks a better expressive power [21–23] that models symmetric relationships of bio-medical KG. Thus, this work employs graph isomorphism network (GIN) [24] and RotatE [22] to distinguish different drug molecular structure and to obtain rich semantic features from bio-medical KG. In addition, MUFFIN directly extracts the nodes embedding from the KG and is limited in obtaining rich potential semantic features on each entity from the KG.

To solve this limitation, this work design a latent feature fusion module to obtain rich latent semantic feature of each drug in KG. In a nutshell, this work presents a novel end-to-end framework called multi-view feature representation and fusion (MuFRF) model, which couples drug molecular structure with bio-medical KG for DDIs prediction. This framework consists of three major building blocks. The first block is multi-view feature extraction and representation, including the graph view feature representation obtained by the graph isomorphism network and KG view feature representation extracted by RotatE. Then, we design a multi-level latent feature fusion block, this block fuses the drugs’ internal (molecular structure) and external (bio-medical KG) feature representation from concatenate-level and scalar-level perspectives. Concatenate-level excavates the latent features from different concatenate operations between molecular structure representation and KG representation. Scalar-level explores the fine-grained latent fusion features using the element-wise add and element-wise product between structure representation and KG representation. And this multi-level architecture further utilizes a multi-head attention module [25] to optimize this multi-granularity latent feature fusion process. The final block is to predict the potential DDIs for binary classification and multi-class classification tasks. Experiments show that MuFRF achieves the highest results for two tasks, this also verifies the integration of molecular structure and semantic information from bio-medical KG is essential. The main contributions of this work can be stated as follows:

- We present a multi-view feature representation and fusion (MuFRF) architecture for potential drug-drug interactions prediction. It can effectively extract the drug molecular structure information and rich semantic features from bio-medical KG.
- Based on the multi-head attention mechanism, we propose a concatenate-level and scalar-level feature fusion method to fuse internal and external features from different granularity operations.
- We deploy extensive experiments on binary and multi-class prediction tasks. The experimental results illustrate MuFRF is superior to classic and state-of-art DDI prediction models.

## Related work

Over the years, some research proposed to predict the potential DDIs by using the drug molecular structure which determines all of its pharmacokinetic (how it is handled by an organism) and pharmacodynamic (how it affects an organism) properties, and ultimately all of its interactions. Vilar et al. [7] utilized molecular structure similarity to identify new DDIs prediction. Cheng et al. [26] combined drug phenotype, treatment, structure and genome similarity with genomic similarity, and sent these similarity features into a HNAI framework for DDIs prediction. Ryu et al. [15] employed the name and structure information of the drug-related component pair to accurately generate important DDI types and outputted them in human-understandable sentences. CASTER [13] developed the sequence pattern mining module which decomposes the SMILES string of drugs into discrete sets of common substructures, improving the performance of DDIs prediction.

These above models both imply drug molecular structure is vital for DDI prediction. However, they ignore rich semantic information in KG constructed by drugs and drug-related entities. Abdelaziz et al. [27] constructed a comprehensive knowledge graph using the drug attributes and the relation among drug-related entities, and developed the drug similarities based on this knowledge graph, and established a linear regression learning model in Apache Spark for predicting DDIs. Karim et al. [17] extracted large-scale DDIs to construct a knowledge graph and trained ComplEx [28] for obtaining the drug embedding features, then the Conv-LSTM network would handle these features for DDIs prediction. Dai et al. [29] first introduced adversarial auto-encoders framework for DDIs prediction. The auto-encoders guaranteed the high-quality negative samples and the discriminator further extracted the embedding of drugs. Meanwhile, Gumbel-Softmax relaxation was employed to solve the vanishing gradient problems. Lin and others [18] constructed a KG capturing semantic relations among entities and employed a graph neural network aggregating more neighbor information to obtain powerful entity embedding representation for DDIs prediction.

Although these KG-based models own good performance, However, they ignore the combination between the drug molecular structure and KG, causing the bottleneck of its predictive ability. In addition, current classical works consider this prediction as a binary classification task, however, it is more valuable but challenging to predict multi-typed DDIs. Thus, MUFFIN adopted MPNN [30] to obtain the molecular structure information from the molecular map and employed TransE [31] for semantic features from the knowledge graph on binary and multi-class classification tasks. The significant difference between our works and this literature is that our method can distinguish different drug molecular structures. Meanwhile, we present a novel latent feature fusion block that can capture not only drug molecular structure information but rich semantic features from the large-scale bio-medical KG.

**Fig. 1:**
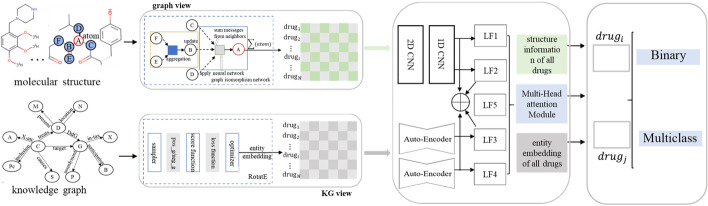
The MuFRF workflow. Feature Extraction and Representation (left part): MuFRF feeds the 2-D molecular graph converted by 1-D SMILES into GIN consisting of message-passing layer and readout layer to learn the graph-view feature representation $h_{G}$. Meanwhile, MuFRF employs the RotatE to obtain the KG-view feature representation $e_{h}$ of entities in KG. Latent Feature Fusion (middle part): we employ concatenate-level and scalar-level strategies to fuse structure information with semantic features in KG, CNNs and auto-encoder further excavate more effective features, and a Multi-head attention module achieves the final latent feature fusion. Classification (right part): the fully connected layer receives the concatenation of latent feature representation and initial graph-based structure representation and KG-based representation to predict potential DDIs

## Methods

Following, we start by modeling drug-drug interaction prediction into supervised binary-class and multi-class classification problems and introduce notations used throughout this paper. Then we present specific procedures of our algorithm.

### Problem formulation

Given $G_{kg}$ representing the semantic features in the knowledge graph and DDI matrix *Y* denoting the molecular structure information for the DDI prediction problem. Our propose is to learn a prediction function ${\hat{y}}_{ij} = F\left( \left( d_{i}, d_{j} \right) \big | \theta ,~G_{kg},~Y \right)$ to judge how likely the drug pair $\left( d_{i}, d_{j} \right)$ is mapped to binary-class or multi-class classification, $\theta$ is our proposed model’s parameter. The specific description of DDI matrix *Y* and knowledge graph (KG) is as follows.

#### DDI matrix

We denote the set of drugs as $D=\left\{ d_{1},d_{2},\ldots ,d_{N_{d}} \right\}$ and the corresponding set of molecular structure diagrams as $G_{drug} = \left\{ g_{1},g_{2},\ldots g_{N_{d}} \right\}$, where $N_{d}$ denotes how many drugs in DDI matrix. For the binary classification task, the DDI matrix *Y* is constructed. *Y* is the set of $y_{ij} \in \left\{ 0,1 \right\}$, $y_{ij} = 1$ indicates that there exists a reaction between the drug $d_{i}$ and drug $d_{j}$. Note that when $y_{ij} = 0$, it does not necessarily mean no interaction between these two drugs in KG, as it may be the potential interaction while it has not been found before. In multi-class prediction tasks, all relation types which are 81 in our DDI pairs are considered for this work.

#### Knowledge graph

In addition to the interactions between drug pairs, we consider the semantic information for drug-related entities (e.g., targets), represented by a knowledge graph. Formally, the knowledge graph (KG) is presented into $G_{kg} = \left\{ (h,r,t) \big | h,t \in E,r \in R \right\}$, each triple $\left( h_{i},r_{i},t_{i} \right)$ indicates there is a relation $r_{i}$ (such as drug-disease, drug-target) between $h_{i}$ and $t_{i}$, where $i \in \left\{ 1,2,\ldots N_{kg} \right\}$, $N_{kg}$ is how many triples exist in the constructed KG.

### Overview

Figure 1 illustrates the overview of the Multi-view Feature Representation of Fusion (MuFRF) framework. MuFRF consists of three modules for DDIs prediction. In the Multi-view feature extraction and representation module, we employ a graph isomorphism network (GIN) to dig the molecular structure information from the molecular map. Meanwhile, we utilize the RotatE to obtain the semantic features from KG (KG refers to bio-medical KG in this work). We design a multi-level strategy in the feature fusion module from concatenate-level and scalar-level perspectives. Concatenate-level uses convolution neural networks (CNNs) to extract latent features based on the different concatenate operations between molecular structure representation and KG representation. Scalar-level utilizes auto-encoder to excavate fine-grained latent fusion features with the operation of element-wise add and element-wise product between structure representation and KG representation. And the multi-level strategy employs a multi-head attention module to optimize this multi-granularity latent feature fusion process. MuFRF obtains the final latent representations of given drug pair $(d_{i}, d_{j})$ in the classifier module. Then we use various classifiers to compute the possibility of DDIs prediction for the binary classification task, And this classifier module outputs the probability score of each relation for the multi-class DDIs prediction. Next, this work will present the detail of the proposed model.

### Multi-view feature representation module

#### Graph-based representation

**Fig. 2:**
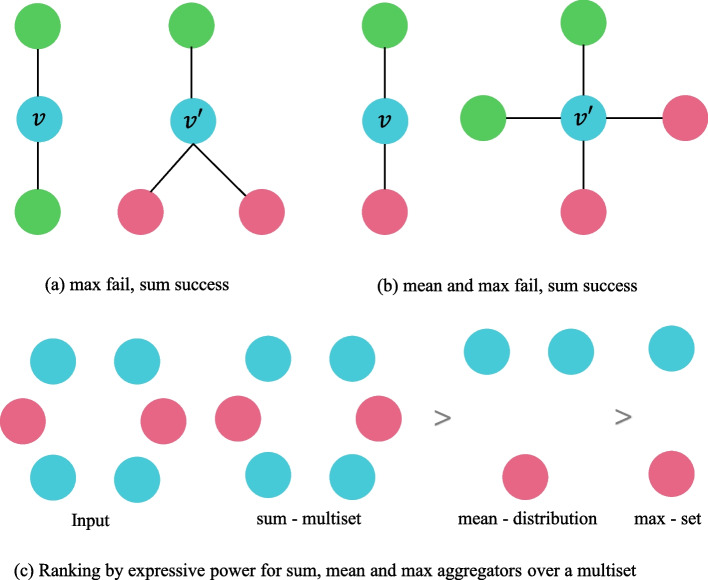
The results of different aggregators. In **a** and **b**, node *v* and $v'$ get the same embedding through max and mean aggregators even though their corresponding graph structures differ, but the sum aggregator can distinguish them. **c** illustrates how different aggregators “compress” different multisets and can give the reasoning that mean and max aggregators cannot distinguish them

The RDKit [32] tool converts SMILES into molecular objects. Next dgl [33] is used to convert molecular objects into bidirectional dgl molecular maps which the existing models can process to extract the structural information of the molecules. The classical methods all use the MPNN [30] framework to extract the structural information of molecules. Still, the methods under the MPNN framework cannot distinguish different graph structures according to the generated graph embedding. Thus, this work adopts the graph isomorphism network (GIN) to generate the structural representation of drugs. Similar to MPNN, it mainly consists of four parts: message function (*M*), aggregation function (SIGMA), update function (*U*), and readout function (*R*). The difference is aggregation, update, and readout functions in GIN are all injective functions, which guarantees GIN can distinguish different drug molecular structures. Figure 2 illustrates this reason.

The message function is the binary message function $u\_add\_e$ of dgl, *u* is the source node, *v* is the target node, *e* is the edge, $u\_add\_e$ operation is to combine the multi-source node features with edge weights and aggregate them into the target node. The aggregation and readout functions are sum aggregators and the update function is a multi-layer perceptron MLP, both of them are injective. Thus, we obtain a GIN framework based on MLP+SUM.

1 $$\begin{aligned} h_{v}^{(k)} = {MLP}^{(k)}\left( \left( 1 + \varepsilon ^{k} \right) \cdot h_{v}^{(k - 1)} + {\sum \limits _{u \in N(v)}h_{u}^{(k - 1)}} \right) \end{aligned}$$

where GIN adjusts the weight of the target node in each iteration through a learnable parameter $\varepsilon ^k$, and merges current node information with aggregated neighbor information to update the current node features. Node embedding from GIN can be applied in node classification and link prediction tasks. For graph classification task, a “readout” function is proposed in this work: the generation of entire graph embedding is derived from the embedding of individual nodes. The READOUT layer uses “concat+sum” to sum all the node features obtained in each iteration to obtain the features of the graph, and then stitch them together. “sum” is to sum the output node representations from all GIN layers.

2 $$\begin{aligned} h_{G} = concat\left( sum\left( \left\{ h_{v}^{(k)} \big | v \in G \right\} \right) \big | k = 0,1,\ldots ,K \right) \end{aligned}$$

**Table 1:** The pattern modeling and inference abilities of several models

Model	score function	symmetry	anti-symmetry	inversion	composition
Structured Embedding	$-\parallel w_{r,1}h - w_{r,2}t\parallel$	$\times$	$\times$	$\times$	$\times$
TransE	$-\parallel h + r - t \parallel$	$\times$	$\checkmark$	$\checkmark$	$\checkmark$
TransX	$-\parallel g_{r,1}(h) + r - g_{r,2}(t) \parallel$	$\checkmark$	$\checkmark$	$\times$	$\times$
DistMult	$<h,r,t>$	$\checkmark$	$\times$	$\times$	$\times$
ComplEx	$Re(<h,r,t>)$	$\checkmark$	$\checkmark$	$\checkmark$	$\times$
RotatE	$- \parallel h \circ r - t \parallel$	$\checkmark$	$\checkmark$	$\checkmark$	$\checkmark$

#### KG-based representation

In this work, consider that the composed KG has various relation types, for instance, symmetry, anti-symmetry, inversion, and composition. Still, the previous TransE, RESCAL, ConvE, and other models cannot solve the above relationship; Table 1 shows the detail. Therefore, in this work, we use RotatE to implement the vector representation of entities and relationships, which is inspired by Euler decomposition: $e^{i\theta } = cos\theta + isin\theta$. Specifically, the embedding is $e_{h} = \left( e_{h}^{(1)},e_{h}^{(2)},\cdots {,e}_{h}^{(d)} \right) \in C^{d}$ for each entity or relationship, *C* is a complex space of dimension *d*. Every element satisfies $e_{h}^{k} = a_{k} + b_{k}i$, $a_{k},b_{k} \in R, k = 1,\ldots ,v$. Here, we give the formula of the score function for a triple (*h*, *r*, *t*).

3 $$\begin{aligned} score(h, r, t) = \parallel e_{h} \circ e_{r} - e_{t} \parallel \end{aligned}$$

We observe the minimum value for this score function is 0, it represents $e_h\circ e_r$ can completely replace $e_t$. The lower the score, the closer the distance to *t* after *h* is rotated by the relation *r* in a complex embedding space, and the greater the possibility that there exists an edge of relation *r* between *h* and *t*. We utilize RotatE to extract multi-relational information because it satisfies all relation types. The strategy of negative sampling has achieved good results in both knowledge graph embedding. Thus RotatE uses a similar negative sampling loss $L_{kg}$:

4 $$\begin{aligned} {log}_{p}= & {} {\ln {\sigma \left( score(h, r, t) - score(h', r', t') - \gamma _{1} \right) }} \end{aligned}$$

5 $$\begin{aligned} L_{kg}= & {} - {\sum \limits _{(h,r,t) \in G,(h', r', t') \notin G}{log}_{p}} \end{aligned}$$

where $\gamma _1$ is a fixed value, $\sigma$ represents the sigmoid function, $(h', r', t')$ denotes the *i*-th negative sampled triple. RotatE embeds the multi-relational information of all drugs through iterative training and then uses it as an input to the feature fusion module to continue mining latent features.

### Feature fusion module

This work adopts a multi-level strategy with a multi-head module to integrate graph-view and KG-view feature representations. The combined latent features own the interactive information on multi-faceted drug features. After the feature extraction and representation module, we initially obtained the structural information $h_G$ and the semantic feature $e_h$ of the knowledge graph. We combine structural information with semantic features using concatenate-level and scalar-level strategies to obtain different latent features. We extract potential features in-depth and then optimize these hidden features using the multi-head attention mechanism. Finally, residual connections cascade the fused hidden features with the initial drug structure information and semantic features. Through the above steps, the final feature representation of all drugs will be sent into MLP to predict the DDI probability score, which can represent whether there is a reaction between two drugs.

#### Concatenate-level

We first conduct different concatenate for initial drug structural information $h_G$ and the semantic feature $e_h$ of the knowledge graph of the drug. Concatenate (dim = 1) means column splicing by row, and concatenate (dim = 0) means row splicing by column. The initial drug structure information and drug semantic features have different dimensions, so the convolution operation is used to make both dimensions 100. Then we make convolution operations on the structural information and semantic features, respectively. Compared with the fully connected layer, the parameter sharing of CNN prevents computing resources wasting, and its translation-invariant nature guarantees the extraction of the location-insensitive information of features. In this work, the structural information of all approved drugs can be expressed as $n*k$, *n* indicates the total amount of approved drugs, where *n* is 2322 and *k* represents dimension (100). All approved drugs are entities in KG, entity Vectors are also represented as $n*k$. The drug’s structural information and semantic features are spliced in rows to capture the feature vector of $2n*k$. The column splicing is performed to gain the feature vector of $n*2k$. Then, 2D CNN makes convolution on these vectors of row and column splicing, and the convolution kernel size is $2*p(10)$. We obtain the matrix vector by combining the drug structure and semantic information as a latent vector. And we continue to input the obtained latent vector into a 1D CNN, the convolution kernel size is *p*(5), and then 1D adaptive average pooling is performed on it, and the latent vectors *LF*1 and *LF*2 with dimension 20 are uniformly obtained.

#### Scalar-level

**Fig. 3:**
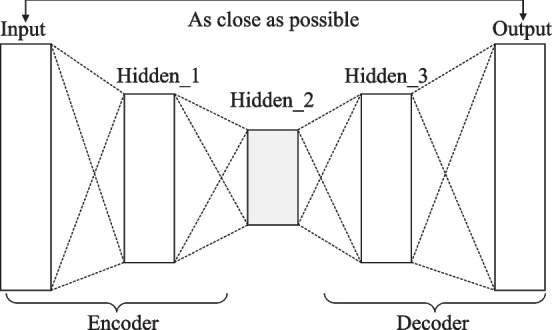
The components of auto-encoder. Hidden_2 is the latent feature we want when the output and the input are as close as possible

We make element-wise add and element-wise product for the initial graph-based and KG-based representation, respectively. Then we utilize an auto-encoder to excavate the latent features. Auto-encoder compresses the given feature vector into a latent-space representation and then reconstructs the target vector. As shown in Fig. 3, it includes two parts: the encoder compresses the given feature vector into a latent space representation. Decoder: This part tries to reconstruct the given feature vector based on the hidden space representation. Our main purpose is to obtain latent vectors. Perform element-wise add and element-wise product operations on the drug structure feature vector and drug semantic feature vector with a dimension of 100 to obtain the feature vector of $n*k$, respectively, and then the auto-encoder will capture the hidden features from the fused feature vector. This auto-encoder has three hidden layers in our model, of which the second hidden layer is the final hidden feature we want, which is represented as *LF*3, *LF*4.

#### Multi-head attention module

**Fig. 4:**
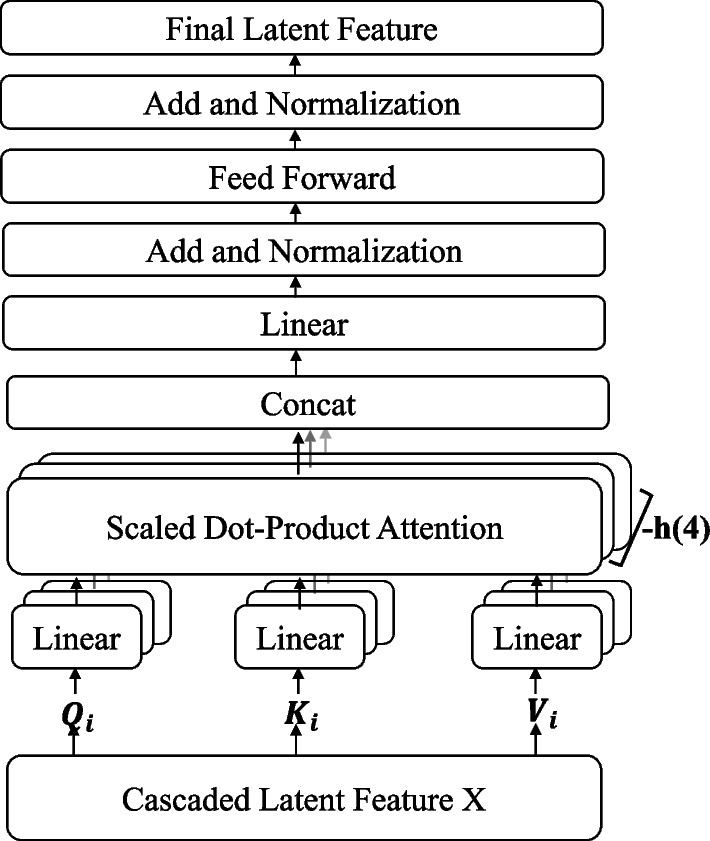
The specific computation operation of cascaded latent feature *X*

Through CNN and Auto-encoder, we obtain four hidden features denoted as *LF*1, *LF*2, *LF*3, and *LF*4. They present different scales of drug structural and semantic features on concatenate-level and scalar-level, respectively. Furthermore, we make element-wise add to capture more latent feature based on these four hidden features, represented by the fifth hidden feature as *LF*5. The hidden features are cascaded and sent to an encoder, which mainly includes scale dot-product attention, add and normalization, and feed-forward operations, shown in Fig. 4. The calculation of this mechanism is mainly as follows.

6 $$\begin{aligned} X_{MultiHead\_ att}= & {} Concat\left( {head}_{1},\ldots ,{head}_{m} \right) W^{o} \end{aligned}$$

7 $$\begin{aligned} {head}_{i}= & {} softmax\left( \frac{Q_{i} \times K_{i}^{T}}{\sqrt{d_{k}}} \right) V_{i} \end{aligned}$$

8 $$\begin{aligned} Q_{i}= & {} X \times W_{i}^{Q} \end{aligned}$$

9 $$\begin{aligned} K_{i}= & {} X \times W_{i}^{K} \end{aligned}$$

10 $$\begin{aligned} V_{i}= & {} X \times W_{i}^{V} \end{aligned}$$

11 $$\begin{aligned} X= & {} Concat\left( (X1,X2,X3,X4,X5),dim = 1 \right) \end{aligned}$$

where *X* denotes the latent feature vector after concatenating *LF*1, *LF*2, *LF*3, *LF*4 and *LF*5, $W_{i}^{Q} \in R^{d_{in} \times d_{Q}}$, $W_{i}^{K} \in R^{d_{in} \times d_{k}}$,$W_{i}^{V} \in R^{d_{in} \times d_{V}}$, and $W^{o} \in R^{{hd}_{v} \times d_{in}}$ are the parameter matrix, $Q_i$, $K_i$ and $V_i$ represent the Q(Query), K(Key), V(Value) matrices. In this work, we take 4 heads attention for the multi-class classification task, $d_{k} = d_{v} = d_{in}/h = 25$. Compared with the total calculated cost of full-dimension single-head attention, the reduction of the dimension of each head guarantees the total calculated cost will not increase.

### Classification

We concatenate the fusion latent feature $X_{MultiHead_att}$ with the initial drug structure feature $h_G$ and the initial drug entity feature $e_h$, where $X_{MultiHead_att}$ denotes local features and global features are $h_G$ and $e_h$. Thus, the global features and local features of all approved drugs can be obtained.

12 $$\begin{aligned} \begin{aligned} D&= \left[{X_{MultiHead_{a}tt} \parallel h_{G} \parallel e_{h}} \right]\\&= \left\{ d_{1},d_{2},\cdots ,d_{i},\cdots d_{j} \right\} \end{aligned} \end{aligned}$$

*D* is the feature representation of all approved drugs. For the DDIs prediction task, we send the final drug feature representation to a dense layer to determine the DDI probability value.

13 $$\begin{aligned} {\hat{y}}_{ij} = \sigma \left( MLP\left( \left[d_{i} \big | \big | d_{j} \right]\right) \right) \end{aligned}$$

${\hat{y}}_{ij}$ represents the probability value of DDIs for binary classification, where $\sigma$ refers to the *sigmoid* function. For a multi-class prediction task, it is the probability score of each relation type, and $\sigma$ is the *softmax* function.

### Training

We minimize the cross-entropy loss to optimize the parameters in the MuFRF framework for binary classification, described as follows:

14 $$\begin{aligned} \begin{aligned} l_{b} =&- ( y_{ij}*log( {\sigma ( {\hat{y}}_{ij})}) \\&+( 1-y_{ij})*log( \sigma ( 1- {\hat{y}}_{ij} ))) \end{aligned} \end{aligned}$$

where $y_{ij} \in \left\{ 0,1 \right\}$ represents whether there exists reaction between drug pair $( d_{i},d_{j})$ for the binary classification. We employ label smoothing cross-entropy loss for multi-class prediction. Label smoothing makes the minimum of the target vector $\varepsilon$. Therefore, the classification results are no longer just 1 or 0 but $1 - \varepsilon$ and $\varepsilon$. The following formula gives the cross-entropy loss function with label smoothing.

15 $$\begin{aligned} l_{m}= & {} (1 - \epsilon )ce(i) + \epsilon {\sum \frac{ce(j)}{N_{c} - 1}} \end{aligned}$$

16 $$\begin{aligned} ce(i)= & {} - {\sum _{i = 0}^{N_{c} - 1}{y(i)log\left( p_{i} \right) }} = - log\left( p_{c} \right) \end{aligned}$$

where $\varepsilon$ is a small positive number (0.15 is selected in the experimental part), *i* is the correct class, and $N_c$ is the number of classes. $p = \left[p_{0},\ldots ,p_{N_{c} - 1} \right]$ denotes a probability distribution, and each element $p_i$ is the probability value that the sample belongs to the *i*-th class. $y = \left[y_{0},\ldots ,y_{N_{c} - 1} \right]$ refers to the one-hot representation of the sample label, when the sample belongs to class *i*, $y_{i} = 1$, and otherwise $y_{i} = 0$. Intuitively, label smoothing places restrictions on the logit value of the right class, making it more approach the logit value of the other classes. Thus, to a certain extent, it is used as a regularization technique and a way to combat model overconfidence. The algorithm of the entire MuFRF training is as shown in Algorithm 1.
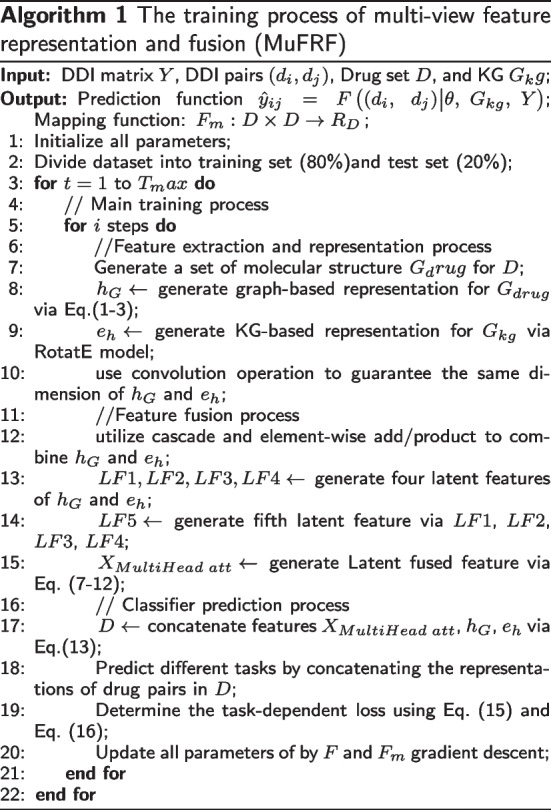


## Results

We mainly present the various experiments to demonstrate the effectiveness of the proposed model in this section.

**Table 2:** Statistic of variables involved in the dataset

Data name	Number
Approved Drugs	2322
KG Entities	96766
KG Relations	105
KG Triples	4488504
DDI (binary-class)	1170940
DDI (multi-class)	172426

### Datasets

#### Binary-class DDIs and KG dataset

DRKG [34] is a comprehensive biological knowledge graph relating genes, compounds, diseases, biological processes, side effects, and symptoms. It is made up of 97,238 entities and 5,874,261 triples. In this work, we capture binary-class DDI data from DrugBank [35] and Hetionet data sets in DRKG, where the relation type is $<ddi-interactions-in:: Compound: Coumpound>$. At the same time, the drugs in the data are all approved, ensuring that the drug has Graph-based embedding information. The number of triples is 1,170,940 pairs. The remaining triples are used as the KG dataset in this work, shown in Table 2.

#### Multi-class DDIs

The DDI multi-relational data was collected from DeepDDI [15], which was extracted from DrugBank [35]. It is made up of 192 284 DDIs and 86 relation types. This work remains 172 426 DDI pairs and 81 relations to stay the same as the KG analyzed from DRKG, eliminating relations with less than 10 samples.

The DrugBank in DRKG only extracts a triplet set of 6 relation types (target, enzyme, carrier, ddi-interactor-in, x-atc, treats) from the original xml format of DrugBank, and part of the binary DDIs dataset is derived from the relation “ddi-interactor-in”, that is, whether there is a reaction to the entity pair. The relationship between entity pairs in the multi-classification dataset extracted from the original DrugBank is divided into 86 relationship types. For example, “label 38 represents the diuretic activity of the latter may be decreased when drug a and b are taken together.”

### Baselines

we compare some representative work with the proposed model.

- DeepWalk [36] takes the sequence generated by the random walk as a sentence and inputs it to the skip-gram algorithm [37] to capture node embedding. And we concatenate Each node’s representation and utilize the classifier to predict DDI.
- LINE [38] is a network embedding model, which combines local and global network structure information to model the node embedding. And this model only uses existing DDIs.
- DeepDDI [15] utilizes SMILES describing the drug chemical structural information to precisely predict important DDI types and outputs them in sentences that humans can understand. It provides guidance for drug development.
- KGDDI [17] trains ComplEx to represent the drug embedding and the authors train the Conv-LSTM [39] network, in which LSTM network can decode global relationships based on features handled by CNN, to predict DDIs.
- KGNN [18] utilizes the rich semantic features from KG and employs a GNN to aggregate neighborhood information for updating the representation of the current entity in the KG.
- MUFFIN [20] combines a message-passing neural network with TransE to capture drug structure representation from the molecular map and semantic features from KG, which guarantees powerful drug representation for DDIs prediction.
- RANEDDI [40] captures the multi-relational information between drug entities in the DDI network and comprehensively considers the original information of neighbors and the information after relationship transformation to obtain the final embedded representation of drugs.
- GRPMF [41] introduces an original regularization strategy to jointly encode prior expert knowledge and graph similarity inference for DDIs prediction.

To validate the significance of each component of MuFRF, we designed seven variants to implement the ablation study:

- MuFRF_ST only employs GINs to extract drug representations with structural features.
- MuFRF_KG uses RotatE to learn the feature representation of each node in the knowledge graph.
- MuFRF-c0 removes concatenate-level (dim = 0) feature to fuse other latent features.
- MuFRF-c1 removes concatenate-level (dim = 1) feature to represent the final drug representation.
- MuFRF-add drops element-wise add operation to obtain the final latent feature embedding.
- MuFRF-p removes element-wise product operations for DDIs prediction.
- MuFRF-attn removes multi-head attention module for feature fusion.

### Evaluation metrics

To test the proposed model performance, the following four performance metrics are needed: the overall classification accuracy (Acc), Precision, Recall, the F1 score. TP indicates true positives, TN means true negatives, FP is false positives and FN represents false negatives.

Acc is the ratio of correctly predicted samples to the total number of samples, Precision is the proportion of correctly predicted interactions among all predicted interactions, and we define the proportion of correctly predicted DDI to the existing DDIs as Recall. In addition, the F1 score is used as a comprehensive criterion. F1 score can be regarded as a harmonic average of model precision and recall. Firstly, label 0 means that there is no interaction between the two drugs. While the potential interaction may has not been found before. We pay more attention on true positive and false negative prediction results to evaluate the performance of models. Here true positive means the model finishes the DDIs prediction correctly and false negative represents the proposed model fails to predict existing drug-drug interaction. In the evaluation metrics, recall is mainly calculated by false negative. Meanwhile F1 is calculated by precision and recall. The more true positive and less false negative, the better the model performance. In this work, besides of accuracy and precision, recall and F1 are the vital metrics to evaluate our proposed model. Their calculation are listed as the following formulas 17-20.

17 $$\begin{aligned}{} & {} \begin{aligned} Acc =(1/N_{c})\sum _{i = 1}^{N_{c}}( {TP}_{i} + {TN}_{i} ) \\ / ( {TP}_{i} + {FN}_{i} + {FP}_{i} + {TN}_{i}) \end{aligned} \end{aligned}$$

18 $$\begin{aligned}{} & {} Precision = \left( {1/N_{c}} \right) {\sum _{i = 1}^{N_{c}}{{TP}_{i}/\left( {TP}_{i} + {FP}_{i} \right) }} \end{aligned}$$

19 $$\begin{aligned}{} & {} Recall = \left( {1/N_{c}} \right) {\sum _{i = 1}^{N_{c}}{{TP}_{i}/\left( {TP}_{i} + {FN}_{i} \right) }} \end{aligned}$$

20 $$\begin{aligned}{} & {} F1 = {(2Precision*Recall)/(Precision + Recall)} \end{aligned}$$

### Experimental setup

This work uses 100-dimensional vectors to represent KG entities, relations and drug structure embedding. For drug molecular map, we adopt the pretrained-GIN to capture the graph-based representation [42]. For KG of drugs, we use the RotatE to model entity and relation representation into complex space. Then, we construct 2D CNN and 1D CNN to dig latent information further for feature fusion component, and their kernel size is set to 10 and 5. we define the hidden size of the last dense layer as 2048. And we determine the output neurons as 1 and 81 on binary and multi-class classification tasks. We design the contrast experiments among MuFRF and baseline models which are shown in Table 3. In addition, we deploy experiments for ablation analysis which also illustrated in Table 3. Moreover, we also design experiments of parameter optimization, the results correspond to Tables 4, 5 and 6, respectively. The experimental learning rate is defined as 0.0015 for binary classification task and 0.001 for multi-class, respectively. And detailed explanation will be given in the parameter analysis section. This work trains model with 200 epochs in Pytorch with Adam optimizer and is performed in an Intel Corel I7 and a GeFore GTX 1080 Ti Graphics Cards. The hyper-parameters of baselines remain unchanged as given in their published work, and these works all employ five-fold cross-validation. We divide this dataset into training set and test set, with the test set accounting for 20%.

**Table 3:** The overall experimental results on baselines, MuFRF, and the ablation study of MuFRF

Method	Binary-class				Multi-class			
	Accuracy	Precision	Recall	F1	Mean-Accuracy	Marco-Precision	Macro-Recall	Macro-F1
DeepWalk	0.8130	0.7970	0.8390	0.8170	0.8000	0.8220	0.7101	0.7469
LINE	0.7810	0.7710	0.8000	0.7850	0.7506	0.6870	0.5451	0.5804
DeepDDI	0.9166	0.9121	0.9241	0.9167	0.8768	0.7986	0.7593	0.7662
KGDDI	0.8926	0.8936	0.8925	0.8925	0.8923	0.7945	0.7667	0.7666
KGNN	0.9034	0.9058	0.8999	0.9029	0.9127	0.8583	0.8170	0.8291
MUFFIN	0.9913	0.9912	0.9913	0.9912	0.9648	0.9568	0.9482	0.9495
RANEDDI	–	–	–	–	0.9707	0.9260	0.9096	0.9161
GRPMF	–	–	–	–	0.9617	0.9627	0.9617	0.9622
MuFRF	**0.9945**	**0.9945**	**0.9945**	**0.9945**	**0.9721**	**0.9765**	**0.9676**	**0.9699**
MuFRF_ST	0.9509	0.9510	0.9509	0.9509	0.9524	0.9344	0.9254	0.9248
MuFRF_KG	0.9200	0.9200	0.9199	0.9199	0.9355	0.8971	0.8641	0.8724
MuFRF-c0	0.9840	0.9840	0.9840	0.9840	0.9705	0.9669	0.9570	0.9594
MuFRF-c1	0.9823	0.9823	0.9822	0.9822	0.9718	0.9754	0.9636	0.9667
MuFRF-add	0.9823	0.9823	0.9823	0.9823	0.9730	0.9684	0.9616	0.9625
MuFRF-p	0.9814	0.9815	0.9814	0.9814	0.9703	0.9662	0.9556	0.9581
MuFRF-attn	0.9821	0.9821	0.9821	0.9821	0.9700	0.9652	0.9574	0.9585

The bold values represent the performance of our proposed model MuFRF outperforms all baselines on each metric for binary and multi-class classification tasks

### Overall evaluation results

The row where MuFRF is located and the preceding row in Table 3 gives the experimental performance of MuFRF and all baselines which have been described above. Compared with all baselines, MuFRF reaches the highest results on each metric for binary and multi-class classification tasks. For example, for these two classification tasks, compared with MUFFIN, the accuracy of MuFRF is improved by at least 0.322%, the precision is increased by 0.332%, the recall and F1 are improved by the same percentage as precision; the mean accuracy of MuFRF is increased by 0.75%, the macro-precision and macro-recall are both improved by at least 2%, the macro-F1 is increased by 2.1%. These findings demonstrate the effectiveness of MuFRF. Note that, DeepWalk and LINE reach a low point compared with other baselines, due to they only predict the known DDIs without considering any drug information. DeepDDI performs relatively less than MuFRF because it adopts structure similarity information as auxiliary information for drug representation. And KGDDI and KGNN models also do not outperform MuFRF, due to they do not excavate the information of molecular structure graph. In the binary-class task, our model exhibits a slight gap than MUFFIN, and in the multi-class classification task, MuFRF has a more obvious advantage than MUFFIN, GRPMF and RANEDDI. MuFRF considers the multi-view feature representation, including KG-view and graph-view, and fully excavates the latent features in comparison with all baselines, which makes it perform well on each metric for these two tasks.

**Table 4:** The impact of different dimensions on baselines, MuFRF and ablation study on MuFRF

Dimension	Binary-class				Multi-class			
	Accuracy	Precision	Recall	F1	Mean-Accuracy	Marco-Precision	Macro-Recall	Macro-F1
32	0.9821	0.9821	0.9821	0.9821	0.9695	0.9596	0.9468	0.9505
64	0.9834	0.9834	0.9834	0.9834	0.9708	0.9659	0.9526	0.9563
100	0.9850	0.9850	0.9850	0.9850	0.9714	0.9659	0.9558	0.9576
128	0.9840	0.9840	0.9840	0.9840	0.9705	0.9669	0.9534	0.9572

### Ablation study

We make an ablation study by comparing MuFRF with its seven variants, and the lower part of Table 3 exhibits the experimental results. MuFRF_ST and MuFRF_KG have relatively low performance compared with other variants that extract molecular structure information and semantic feature in KG. Moreover, MuFRF_ST has a better score than MuFRF_KG, which verifies the molecular structure information is vital for DDI prediction. Other variants show concatenate (dim = 0 or dim = 1), element-wise add, and element-wise product bring some improvement to all evaluation criteria in binary and multi-class classification tasks. MuFRF-attn shows that there is an inevitable loss in two different prediction tasks in comparison with MuFRF, which verifies that the attention mechanism optimizes the feature captured by this multi-level strategy again.

**Fig. 5:**
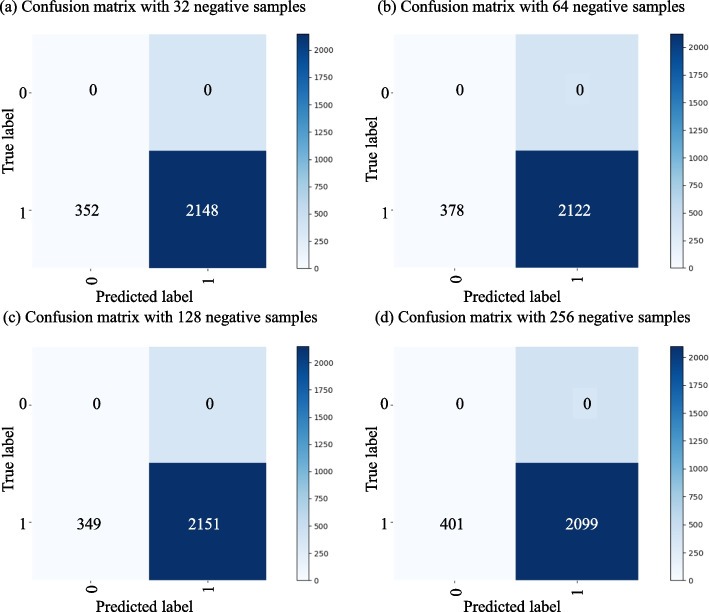
Confusion matrix with different negative samples on binary-class prediction task

**Fig. 6:**
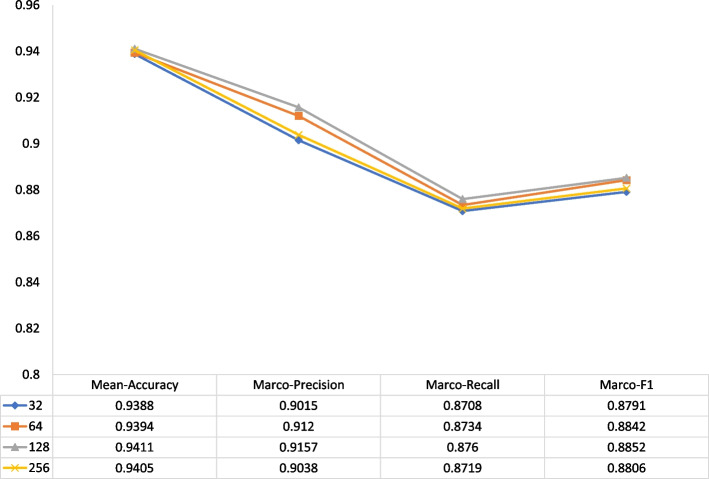
The impact of negative samples of different sizes on performance for multi-classification tasks

In a nutshell, the MuFRF model outperforms all baselines and all variants of MuFRF, which completely implies that integrating Molecular structure information and semantic feature in KG is vital in all prediction tasks. And the proposed multi-level latent feature fusion between structure information and semantic features is essential for DDIs prediction.

### Parameter analysis

Here, we will study whether the varieties of key parameters influence the performance of MuFRF. To show how different parameters will affect the model performance, experimental results on each metric are given for binary and multi-class classification tasks. The specific experiment results and analysis are as follows.

#### Impact of negative sample size

Figures 5 and 6 illustrate the effect of different negative triplets on each positive triplet during KG training. For the binary classification task, we fix the evaluate batch size as 2500 and exhibit the influence with the confusion matrix. In comparison with 32, 64, 256 sample size, each evaluation criterion of MuFRF reaches the peak when the sample size is 128. Line graphs plot the performance of different negative sample sizes across all metrics for the multi-class classification task. We can see that MuFRF obtains more valuable information with enough negative samples. However, there exists more noise in the KG representation process when the negative samples increases, this will be further investigated in future work.

**Table 5:** The selection of *n*-heads for DDIs prediction task

*n*-heads	Binary-class				Multi-class			
	Accuracy	Precision	Recall	F1	Mean-Accuracy	Marco-Precision	Macro-Recall	Macro-F1
1	0.9832	0.9833	0.9832	0.9832	0.9706	0.9582	0.9497	0.9512
2	0.9850	0.9850	0.9850	0.9850	0.9708	0.9606	0.9512	0.9532
3	0.9842	0.9842	0.9842	0.9842	0.9710	0.9574	0.9484	0.9499
4	0.9831	0.9831	0.9831	0.9831	0.9714	0.9659	0.9558	0.9576
5	0.9822	0.9822	0.9822	0.9822	0.9712	0.9647	0.9504	0.9540

**Fig. 7:**
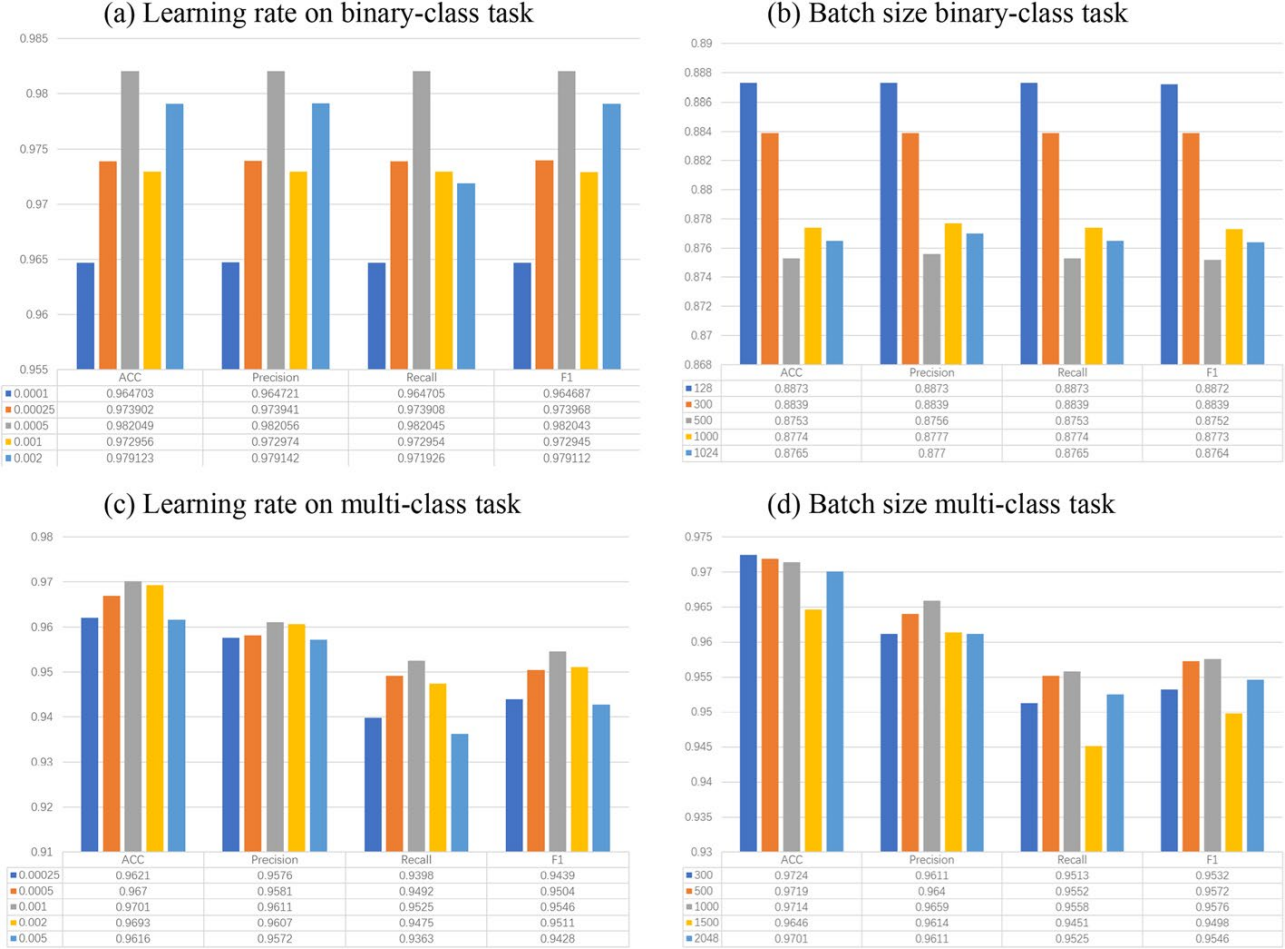
The impact of the hyper-parameters

#### Impact of the embedding dimension

How multiple embedding dimensions influence model performance is shown in Table 4. Specifically, this work investigates the effect when the embedding dimensions are changed from 32 to 128. The performance on MuFRF rises with increasing the embedding dimension from 32 to 100. Still, when the embedding dimension is 128, the performance declines compared to the previously selected dimension. Thus, we fix the embedding size as 100 dimensions in our experiment.

#### Impact of the *n*-heads

To verify how the selection of heads in the multi-head attention module influences each evaluation criterion on these two tasks, we conduct experiments with different selections of *n*-heads. We have fixed the embedding size at 100 and the epoch at 50, respectively. Table 5 shows we employ two heads for binary classification and four heads for multi-class classification, the results on all criteria are optimal. Thus, in this work, we select two or four heads for the DDIs prediction to train the best model.

**Table 6:** The selection of smooth value in multi-class loss function

smooth value	Acc	Precision	Recall	F1
0.0	0.9581	0.9502	0.9405	0.9415
0.1	0.9623	0.9557	0.9428	0.9459
0.15	**0.9670**	**0.9581**	**0.9492**	**0.9504**
0.2	0.9630	0.9571	0.9404	0.9446
0.25	0.9632	0.9507	0.9401	0.9417
0.3	0.9637	0.9563	0.9493	0.9493

The bold values represents when the smooth value is 0.15, the performance of our model reaches the best on all criteria for the multi-class classifcation task

**Table 7:** Predicted DDI types of drug pairs

Drug a	Drug b	Interactions type	Evidence
Selexipag	Epoprostenol	Drug a may increase the hyperglycemic activities of Drug b	DrugBank
Selexipag	Fluconazole	Drug a may increase the neuromuscular blocking activities of Drug b	Unconfirmed
Vorapaxar	Amiodarone	Drug a may decrease the sedative activities of Drug b	DrugBank
Vorapaxar	Argatroban	The metabolism of Drug b can be decreased when combined with Drug a	DrugBank

#### Impact of the hyper-parameters

To guarantee our model could achieve good performance, learning rate and batch size are critical. We first determine the learning rate for the binary classification task, where we set the epoch and batch size as 50 and 1024, respectively. Figure 7a shows when the learning rate is 0.0005, the results on all criteria reach the peak. Thus, we identify the learning rate as 0.0005. Then, we only set the epoch to 1 to quickly train our model with different batch sizes. Figure 7b illustrates the results on all criteria are best when the batch size is 128. However, considering the number of our train data is 1873504, a small batch size of 128 costs too much time, thus we maintain the original batch size of 1024. As we all know, the learning rate setting should be proportional to the setting of batch size, which is the so-called linear scaling rule. And literature [43] explains this rationale. As shown in Fig. 7, the results of all standards are the best when the batch size is 128. However, They take too much training time on the experimental deployments. To reduce the training time, we design several experiments to find relations among batch size and performance and time cost. According to the experimental results shown in Fig. 7, we employ larger batch size such as 1024. The model performance still reaches 87.64 on F1 score when the batch size is 1024, then we will continue to increase our batch size linearly, the final batch size is 3072 due to the memory size of our lab server. The Linear Scaling Rule determines that as the batch size increases by a factor of *k*, the learning rate also increases by a factor of *k*. Finally, we determine the batch size as 3072, thus, the learning rate should be 0.0015 in this work for the binary classification task. For the Multi-class classification task, Fig. 7c and d present that scores of all criteria are highest with a learning rate of 0.001 and batch size of 1000.

#### Impact of the smooth value

We employ the label smooth cross entropy function for the multi-class classification task. When the smooth value is 0, this loss function is also the standard cross entropy function. To avoid over-fitting and alleviate the impact of wrong labels, this work assigns the smooth value a small constant. We have fixed the learning rate of 0.001 and batch size of 1000. Six smooth values, which are 0.0, 0.1, 0.15, 0.25, and 0.3 are given, and 50 epochs are performed to train the prediction model. Table 6 indicates when the smooth value is 0.15, the results in all criteria are the best. Thus, the best prediction model is implemented when we fix a learning rate of 0.001, a batch size of 1000, and a smooth value of 0.15.

## Discussion

This work will test the practical value of MuFRF and discuss the real application of MuFRF through the case study. Table 7 illustrates the predicted results for two common drugs, Selexipag and Vorapaxar. Selexipag is a non-prostanoid IP prostacyclin receptor agonist and it is taken for treating pulmonary arterial hypertension. Vorapaxar is a platelet aggregation inhibitor for reducing thrombotic cardiovascular events in patients with a history of myocardial infarction (MI) or peripheral arterial disease (PAD). For these drug pairs, we try to discover evidence for supporting them from DrugBank, PubMed, and Drug Interactions Checker tool provided by Drugs.com. Table 7 shows that some of the DDI pairs have evidence, that can signify the effectiveness of MuFRF. For the utterly new DDIs predicted by MuFRF, we expect to provide certain guidelines for future exploration and experimental validation.

## Conclusions

this work develops a multi-view feature representation and fusion (MuFRF) framework to achieve drug-drug interactions prediction on both binary-class and multi-class classification tasks. MuFRF designs a multi-level latent feature fusion strategy and uses a multi-head attention block to fully exploit and optimize the latent feature from the graph view and KG view. Ablation study can demonstrate the multi-level feature fusion between structure information in the molecular graph and semantic features in bio-medical KG is effective. Moreover, the attention mechanism can effectively optimize the latent feature of all drugs. Compared with baselines on DDIs prediction, experiments show that our model is effective on two real-world datasets. This work mainly focuses on binary classification and multi-class classification tasks. However, there exists a multi-label phenomenon for DDIs pairs in TWOSIDES [10] from DRKG. Thus, we will further investigate multi-label classification in future work.

## Data Availability

The datasets used and analysed during the current study available from the corresponding author on reasonable request.

## Abbreviations

MuFRF: Multi-view feature representation and fusion
DDIs: Drug-drug interactions
ADRs: Adverse drug interactions
KG: Knowledge graph
GIN: Graph isomorphism network
CNNs: Convolution neural networks
SMILES: Simplifed Molecular Input Line Entry System
MLP: Multi-layer perceptro
